# The Cat Desexing Policies and Activities of Private Veterinary Practices in Queensland

**DOI:** 10.3390/ani10050841

**Published:** 2020-05-13

**Authors:** Mandy B A Paterson, Michael O’Donoghue, Philip Jamieson, John M Morton

**Affiliations:** 1 Royal Society for the Prevention of Cruelty to Animals, Queensland, Wacol, QLD 4076, Australia; gaphjam@gmail.com; 2 People & Pets, Oxley, Qld 4075, Australia; mike@petsandpeople.com.au; 3 Jemora Pty Ltd., Geelong, VIC 3220, Australia; johnmorton.jemora@gmail.com

**Keywords:** cats, desexing, veterinarians, pre-puberty, over-population

## Abstract

**Simple Summary:**

The Royal Society for the Prevention of Cruelty to Animals (RSPCA) shelters around Australia take in hundreds of unwanted adult cats and kittens each year. Widespread desexing (or sterilizing) of cats before they have a chance to become pregnant would reduce numbers of kittens born, and hence, would be expected to reduce numbers of cats entering shelters. Veterinarians have an important role to play in promoting desexing of cats before they reach puberty, and this paper reports on a survey conducted to describe Queensland veterinary practice policies and activities. We ascertained ages that veterinarians recommended for desexing of cats, ages at which desexing actually occurs, what veterinary practices are doing to promote desexing, and what the respondents see as the barriers to desexing before puberty.

**Abstract:**

Cats are prolific breeders, and if most cats were desexed prior to puberty, numbers of unwanted cats and kittens, and hence numbers entering shelters, would be expected to decline. Although traditionally in Australia it has been reported that 90% of veterinary clients’ cats are desexed, there are still hundreds of cats and kittens that end up unwanted and in shelter care annually. In this study, we surveyed Queensland veterinary practices to describe ages that veterinarians are recommending cats should be desexed at, ages at which desexing actually occurs, what veterinary practices are doing to promote desexing of cats, and what veterinarians see as the barriers to desexing of cats before puberty. A questionnaire was developed and sent to all veterinary practices in Queensland. The response rate was 50%. Almost 45% of respondents recommended desexing at the traditional age of 6 months, which is later than puberty in most cats; for more than 56% of practices, the actual average age at which desexing occurred was at least 6 months; and in a substantial proportion of practices, when desexed, high percentages of cats had already had litters. Most practices took steps to encourage their clients to have their cats desexed, and most thought these steps were effective. The results from this study suggest that although veterinarians generally agree that cats should be desexed prior to having their first litter, recommended and actual desexing ages are commonly too late to ensure this is achieved. Better understanding is required about both the likely impact of more veterinary practices recommending and conducting desexing before puberty on numbers of unwanted cats and numbers surrendered to shelters, and the drivers of age at which cats are desexed. This could inform strategies to reduce numbers of unwanted cats.

## 1. Introduction

Cat overpopulation occurs where there are more cats in the community than homes to accommodate them [1,2], and it remains a significant concern globally [3,4]. Desexing (or surgical sterilization; i.e., castration and ovariohysterectomy) is an important tool [2,4,5], indeed, one of the principal tools [6,7], in efforts to reduce cat overpopulation. Studies suggest that over 90% of owned cats in Australia are desexed [8,9]. However, evidence from the United States suggests that some 20% of female cats have litters before they are desexed [10]. Moreover, one study found that the mean numbers of litters born to female pet cats before being desexed was only a little less than that for the lifetimes of female pet cats that were never desexed [4]. A study of cat admissions to Australian Royal Society for the Prevention of Cruelty to Animals (RSPCA shelters found the percentage of admissions where the cat had already been desexed to be relatively low; only 36% of all feline admissions were previously desexed (50% of adult cats and 22% of kittens), and only 47% of owner-surrendered cats were previously desexed [11]. Of cats entering Australian RSPCA shelters in Sydney, Melbourne, and Brisbane, unwanted kittens contributed substantially to intake, representing 19% and 29% of owned and unowned cats, respectively [12]. The term “spay delay” has been coined to describe when sterilization surgery is delayed [13]. Traditionally, cats were not desexed until they were at least 6 months of age [14,15,16]. However, female cats can reach sexual maturity from as early as 3.5 months of age (i.e., 15 weeks) [17,18] and male cats are fertile from about 5 months of age [17]. The resultant “spay delay” may be playing an important role in maintaining cat overpopulation. A Western Australian study concluded that desexing prepubescent female cats could make a substantial difference to cat welfare, by reducing the numbers of kittens surrendered to shelters [8]. Prepubertal desexing is defined as desexing undertaken at any time prior to the onset of puberty (sexual maturity), usually around 4 months of age. This concept encompasses but is distinct from early age desexing. Definitions of early age desexing vary in the literature, but it has been described as desexing at as early an age as is safely possible, traditionally 8–12 weeks of age [19]. Thus, using this definition, prepubertal desexing includes early age desexing but also desexing at older ages prior to puberty. It is beyond the scope of this paper to provide a detailed review of the effects of prepubertal desexing on cats. However, based on the majority of the scientific literature, prepubertal desexing of cats is a safe tool (no less safe in terms of effects on health and behaviour than traditional desexing at 6 months of age) to help prevent unwanted litters [16,20,21].

It is mandatory to desex cats in some Australian states and territories [22], but only in the Australian Capital Territory (ACT) do the provisions require the desexing of cats before the traditional 6 months of age. Section 74 of the Domestic Animals Act 2000 (ACT) (as amended in 2007) states it is an offence to own an undesexed cat that is 3 months of age or older without a permit. However, the Australian Veterinary Association (AVA) considers that compulsory desexing of privately owned companion animals has not been shown to substantially reduce cat overpopulation [5]. The AVA policy is that surgical desexing is an important tool to reduce the number of unwanted companion animals, particularly when combined with community education programs, and that veterinarians should make decisions about age of desexing on a case by case basis in consultation with the owner [5]. In the United States (US), the American Veterinary Medical Association (AVMA) endorses the view that cats not intended for breeding should be desexed by five months of age [23]. 

Veterinarians are a key source of information for owners on cat health and so their attitudes towards and behaviours in the promotion of desexing, specifically prepubertal desexing, are vital to ensuring the effective use of cat desexing to prevent cat overpopulation [10]. To that end, it is important to know the attitudes and behaviours of veterinarians towards these procedures. This involves understanding not only the ages at which they recommend desexing should occur by, but also by what ages it actually occurs in their practice, and what steps they take to promote the procedure.

Numerous surveys have been undertaken to describe veterinarians’ attitudes and behaviours in relation to cat desexing [19,24,25,26,27,28]. A survey of New York State veterinarians [24] found that the median earliest recommended age for desexing was 5.5 months for female cats and 6 months for male cats. While more than three quarters of respondents believed that prepubertal desexing would help reduce pet overpopulation, they expressed concerns about perceived anesthetic, surgical, and perioperative complications of prepubertal desexing. However, respondents were not perpetuating erroneous ideas about desexing, such as that female cats should have a litter before being desexed. Similarly, a United Kingdom (UK) survey [25] found that while the mean age recommended by veterinarians for desexing cats was 22.6 weeks, 51% of veterinarians recommended client-owned cats be desexed at six months of age or older and only 28% considered it appropriate to desex 12 to 16-week-old kittens. Moreover, although some owners requested that their cats be desexed younger than six months of age, 37% of veterinarians reported that they had declined to desex a clinically healthy cat 12 to 16 weeks of age and 10% had declined to do so with respect to 17 to 20-week-old cats. There is evidence that the age at which desexing actually occurs varies between the UK, New Zealand and Australia, with veterinarians in New Zealand and Australia being more likely to desex cats between 3.5 months and 4 months of age than veterinarians in the UK [26]. In a later study of Australian veterinarians [27], desexing was most commonly recommended at the traditional age of 6 months with a mean recommended age for desexing cats of 20.8 weeks. Veterinarians who recommend desexing non-breeding cats before they have an estrus or litter still tend to prefer to desex cats when they reach at least 4 months of age [26,27]. Somewhat surprisingly, it appears that veterinary nurses and veterinary students are more conservative with their opinions on the best age to desex than practicing veterinarians [28], usually recommending an older age. 

It appears from these studies that there is no general consensus amongst veterinarians about when cats should be desexed, particularly in relation to the use of prepubertal desexing. Cat desexing also requires that owners be willing to have the surgery carried out. One study in the United States found approximately 20% of cats owned by the respondents of a phone survey were not desexed by choice [7]. The most common reason was the belief that the cat was confined and therefore did not need to be desexed. This study [7] did not find cost as a major factor in the decision not to desex the cat, whereas other studies have found cost to be important [13].

Given the overall lack of a general consensus, this study extends on previous studies in surveying private veterinary practices in Queensland, Australia, in 2017. The aims of the survey were to describe the ages at which Queensland veterinarians recommend that cats be desexed and the actual ages at which it is occurring, to describe how veterinary practices are promoting desexing, to ascertain what veterinarians see as the barriers to desexing of cats before puberty, and to assess the perceptions of veterinarians about the effectiveness of their practice promotions. To the best of the authors’ knowledge, this survey was the first to explore in detail, ways that veterinary practices promote desexing, and perceptions of their effectiveness. It is hoped that the results obtained by the survey might inform policies and practices of the RSPCA, other private and government agencies, the AVA, and Australian veterinary practices in relation to cat desexing, with the aim of encouraging the wider use of prepubertal desexing where appropriate.

## 2. Materials and Methods 

This research was a collaboration between RSPCA Queensland (RSPCA Qld) and the AVA, Queensland branch (AVA Qld). The research was conducted in accordance with the *National Statement on Ethical Conduct in Human Research (2007).* A questionnaire was developed using Survey Monkey^®^ to collect data to address the above aims. It was created in April 2016, modified in March 2017 after testing with a number of veterinarians, and then made available from April 2017 to September 2017. The questionnaire included multiple choice and free text questions. The RSPCA Qld maintains a database of all veterinary practices in Queensland. In April 2017 there were 517 veterinary practices on the RSPCA Qld database.

An email invitation was sent from RSPCA Qld to every Queensland veterinary practice on the database to participate in the survey with a link to the online questionnaire. The invitation explained the aims of the survey and asked that one person complete the survey on behalf of the practice. The questionnaire may have been completed by the head or another veterinarian, practice manager, head nurse, or other interested party. Only one response per practice was requested and this was partially ensured by setting the online software so that the questionnaire could be completed only once from any particular computer. A reminder email was sent a month later.

The questionnaire consisted of 12 questions, including two demographic questions (age category and gender of the respondent, but not their role in the practice) (Appendix A). Some of the remaining 10 questions were worded to specifically collect practice-level information, while others collected the respondents’ views. These 10 questions asked how important the respondents thought the role of veterinarians is in terms of helping reduce cat overpopulation, the recommended age that cats be desexed, the average actual age that cats are desexed at the practice, what steps the practice takes to promote desexing, and how successful the respondent felt these various steps are. They were also asked what they saw as the barriers to desexing and how many of the female cats they desexed had already had a litter. The questions on age of desexing (recommended and actual) did not provide the opportunity to answer a different age according to gender. Additionally, the opportunity to provide a different age for owned and rescue animals was not provided.

Two possible promotional steps (“Suggest desexing when new cat receives final vaccination” and “Suggest setting a desexing date when the cat receives its final kitten vaccination”) were combined for some analyses; the combined variable was set as “yes”; if the respondent selected either or both of these options. The correlation between recommended desexing age and actual age that desexing occurred was assessed using Spearman’s correlation coefficient using just the 224 practices that nominated single age categories for each of recommended and actual ages. The spearman and ci2 commands in Stata (version 16, StataCorp, TX, USA) were used. The 95% confidence interval was based on Fisher’s transformation. *p*-values for the comparison among respondents that used the various steps to promote desexing of the recommended and actual desexing ages were calculated using Fisher’s exact tests performed using Stata’s tabulate command. Not all respondents answered all questions; numbers of responses are indicated with results. The number of respondents used for each analysis is shown. For some analyses, percentages do not sum to 100% due to rounding.

## 3. Results

Two hundred and fifty six responses were obtained, representing a response rate of 50%. Not all responses included answers to every question. Eighty-one percent of respondents (206/253) considered private practice veterinarians to have a very important role in preventing the birth of unwanted kittens, with 17% (43) selecting somewhat important and 2% (4) selecting not important. 

Forty five percent of respondents recommend the desexing of cats at the traditional age of 6 months and for more than 56% of practices, on average, cats were not actually desexed until 6 months or more of age (Table 1). Only 8% of respondents recommend desexing of cats at 16 weeks of age or less (a category where virtually all would be prepubertal) and even fewer practices, less than 5%, actually desexed on average at 16 weeks of age or less. The questions did not allow respondents to provide different ages for male and female cats, nor for owned and rescue cats; these results cannot report if there were differences according to gender or animal source. Veterinarians selected the “other” option (an alternative answer offered in several of the questions, see Appendix A) when, for example, they had different age recommendations for male and female cats or for clients’ cats and “rescue” cats, or they recommend an age range that did not align with the options provided. The “other” option for the actual age question again included that they desex client and “rescue” cats at different ages, and they wanted to report an age range different from the ones supplied. Others reported being unsure, that they had more than one common age (for example, 6 and 12 months), or that there was no common age.

In response to the question, “Does your practice take active steps to promote desexing/remind clients to have their pet desexed?”—241 respondents selected one of the three multiple choice options offered. Of these, 92% (221/241) selected yes, 6% (15) selected intermittently, and 2% (5) selected no. Percentages of practices that used various specific steps are shown in Table 2. In 84% of practices, desex options were discussed with the owners of all entire adult cats. In 96% of practices, the procedure was discussed with owners when they brought their kittens in for vaccination. It was very common to suggest the procedure (73%), or the setting of an appointment for the procedure (60%), when a cat was given its final vaccination. Reminders were common tools—by phone/email/text/post to set a desexing appointment (69%) or as a reminder the day before surgery (67%).

Amongst other steps taken, in 57% of practices, information on desexing was available on the practice website; information on recommended age for desexing was provided by 56% of practices on their websites, on information sheets, or by way of advice from reception staff. Less commonly used encouragements were incentives (such as free microchipping) (17%) or reduced pricing for the procedure if carried out before the cat’s first estrus (21%). 

Most respondents considered the steps their practice was taking to promote the desexing of their clients’ cats were either successful (67% or 162/243) or very successful (27% or 65/243).

After combining “Suggest desexing when new cat receives final vaccination” with “Suggest setting a desexing date when the cat receives its final kitten vaccination”, there were 12 possible steps (including “other”) that the practice could use to promote desexing/remind clients to have their pet desexed. Most practices (75% or 184/244) used 4–8 steps, with 17% (42) using 0–3 steps, and 12% (30) using 9–11 steps.

Respondents could select from a pre-specified series of reasons why people do not have their cats desexed prior to puberty (Table 3). The most commonly perceived reason was that the owner could not afford (or think they could not afford) the procedure (74%), and 52% of respondents considered that their clients thought it was too expensive. Simply forgetting about it or not getting around to it was identified by 68% of respondents. A range of other considerations were also identified. While these include considerations relating to the cat’s welfare—that it is best to allow the cat to have a litter before desexing (nominated by 40% of respondents), the cat being too young (23%), and that the operation is dangerous (12%)—they also include considerations of convenience, such as that it is nice for their children to see the pet have a litter (39%), the litter can be sold (26%), and the belief they can confine the cat sufficiently to prevent a litter (27%) (Table 3).

In a substantial proportion of practices, high percentages of cats desexed had already had a litter. More than 25% of cats desexed had already had a litter in 11% (25/238) of practices, 11–25% in 28% (67/238) of practices, 1–10% in 59% (141/238) of practices, and none in only 2% (5/238) of practices.

Average actual ages that cats are desexed are shown in relation to recommended desexing age in Table 4. There was only a moderately close correlation between recommended and actual age (Spearman’s correlation coefficient 0.65; 95% confidence interval 0.56 to 0.72). For 64% of practices, the actual age was as recommended by that practice (144 of the 224 practices that nominated one of the age categories offered for each of recommended and actual ages). The most common discordance was the actual age being older than recommended (30% of practices; 68/224). In contrast, the actual age was less than that recommended for only 5% of practices (12/224). Of the 113 practices that recommended desexing at 25 weeks or less, for the majority (60% or 68), actual desexing age was older than recommended. In contrast, of the 205 practices that recommended desexing at 17 weeks or older, for only 12 (6%) was the actual age less that that recommended.

In total, 235 respondents nominated one of the age categories offered for recommended desexing age. Percentages of these that used the various steps to promote desexing are shown with respect to recommended desexing age in Table 5. Percentages of respondents that sent appointment reminders before desexing surgery varied with recommended desexing age, with the highest percentage where the recommended age was 21–25 weeks and the lowest where the recommended age was over 25 weeks. Percentages of respondents that had recommended ages for desexing available were lowest where the recommended age was 20 weeks or less.

In total, 239 respondents nominated one of the age categories offered for actual average age that cats are desexed. Percentages of these that used the various steps to promote desexing are shown with respect to actual age that cats are desexed in Table 6. Percentages of respondents that “discuss desexing with new owners when they bring their kitten in for vaccination” was highest for cats desexed between 21 and 25 weeks and lowest for kittens up to 16 weeks. This was also true for “send a desexing reminder by email/sms/phone, etc., if an appointment is not made for the new pet,” and “send an appointment reminder the day before desexing surgery has been booked in.” However, the highest percentage for “have recommended ages for desexing available: on website; on information sheets; and as advice from reception staff” was highest for cats desexed over 25 weeks of age and lowest (0%) for up to 16 weeks of age.

## 4. Discussion

Fournier and Geller [7] describe cat overpopulation as a societal problem and suggest a framework to help to humanely solve the problem. They argue that environmental factors such as ineffective shelter policies and inaccurate depictions of animals by the pet industry are the main factors in need of change. They do not mention the role of private veterinary practitioners. In a Western Australian study, the authors concluded that desexing prepubescent female cats could make a substantial difference to cat welfare by reducing the numbers of kittens surrendered to shelters [8]. However, as a cat’s reproductive status ultimately depends on its owner’s willingness to have it desexed [29], promoting cat desexing, and in particular, prepubertal desexing, has been recently described as the most important method of controlling cat overpopulation [6,7,8]. The vital role of veterinarians in promoting the effective use of cat desexing in the community is recognised by the veterinary community [10,25,27,28] and by more than 80% of respondents of this survey who considered private veterinary practitioners to have a very important role in preventing the birth of unwanted kittens. Australian veterinarians on the whole appear to be more supportive of prepubertal desexing than those in New Zealand and the United Kingdom, not only in recommending prepubertal desexing but also in actually undertaking it [26].

In the current study, almost 45% of respondents recommended desexing of cats at the traditional age of 6 months with only 8% recommending 16 weeks of age or less, and 16% recommending 17–20 weeks. This is consistent with results from earlier studies in Australia [19,27] and elsewhere; for example, the United Kingdom [25] and the United States [24]. Results from these earlier studies indicate that veterinarians are generally in agreement with the benefit of prepubertal desexing in terms of cat overpopulation control (but not necessarily early age desexing) [19,26,27]. However, means of ages at which veterinarians from Australia, New Zealand, and the United Kingdom believe puberty typically occurs in cats were 5.7–5.9 months [26], much older than the age when puberty can occur in cats [17,18]. Therefore, even though these veterinarians support desexing before puberty, their recommended desexing ages, and the actual ages that they desex cats at may not be consistent with that. There seems to be a general view amongst veterinarians that desexing at ages younger than 5 months is appropriate for cats in shelters and in care of welfare groups but not for private clients [24,25]. This belief was shared even by veterinarians who believe that there are anaesthetic and surgical risks to desexing at an early age [25]. Thus, the advantages of ensuring cats are desexed before adoption are perceived as outweighing any adverse effects of desexing before puberty. This was also reflected in some comments included in the present study. The principle that different ages are appropriate for owned and shelter cats is taught at some veterinary schools [30]. If desexing cats younger than 5 months increases the risks of adverse outcomes, those risks are no less for shelter cats than owned cats. Therefore, older desexing ages for owned cats can be justified only if conception can be prevented in such cats or if one believes that birth of unwanted kittens is of little consequence.

Previous exposure to early age desexing and the perception that there are too many kittens in a practice area increases the likelihood that a veterinarian will recommend prepubertal desexing of clients’ cats [25]. Veterinarians have expressed concerns about increased anaesthetic risk in cats undergoing prepubertal desexing [19,26,27] although results in the scientific literature do not appear to support these concerns and suggest that veterinarians should desex cats at an earlier age than their current recommendation [27]. Better understanding by veterinarians of the safety of desexing before puberty is desirable and also that they do not perpetuate erroneous ideas about neutering raised by clients, such as, it is better for cats to have a litter before being desexed [24]. An independent systematic detailed critical review of the scientific literature may be important to allow veterinarians to assess whether prepubertal desexing of cats poses additional anaesthetic risks over traditional age desexing and/or increases the risks of adverse health and behaviour outcomes. In a short review, Kustritz concluded that no significant short-term or long-term effects had been reported for cats desexed under 12 weeks of age compared with traditional age desexing [31]. In a study of 1660 cats published in 2004, those authors reported that cats desexed when aged under 5.5 months developed no serious medical or behavioural issues compared with cats desexed at older ages [32]. However, an updated systematic critical review of all relevant evidence collectively may be warranted.

In the present study, we also examined the actual average ages that cats were desexed and compared this to the recommended ages. There was only a moderately close correlation between recommended and actual average age of desexing and the most common discordance was the actual average age being older than recommended (30% of practices). This is consistent with results from a United States survey of veterinarians where over 30% of respondents recommended a younger age than they actually practiced [24]. In our study, we do not know whether this discordance was occurring because veterinarians were actively desexing at a later age than they recommended, or whether the later ages were due to the cat owners not making appointments as early as recommended by respondents. A study in the United Kingdom found that owners tended to have their cats desexed at the age they believed appropriate and that “action” was linked to a “plan” [33]. This link appeared to be true even for cats that had not previously been presented to a veterinarian. The study did not examine what contributed to the plan, but it appears that owners generally have a poor understanding of feline reproduction [6,10,34]. More and better engagement between veterinarians and owners, and targeted educational programs, could have positive results in changing the actual desex ages to those recommended by the owner’s veterinarian [6,29,33]. A New Zealand study reported that educational programs have a positive effect on owner attitude to desexing [29].

Another factor influencing the age of desexing may have been how actively the veterinary practices promoted desexing and the use of reminders. In the current study, almost all practices (98%) took active steps to promote desexing and almost 70% either sent an appointment reminder the day before surgery had been booked and/or a reminder to make an appointment to have their cat desexed. In more than 96% of practices, the procedure was discussed with owners when they brought their kittens in for vaccinations. This is in line with recommendations suggested elsewhere [24]. Most respondents believed the steps they took to promote desexing were effective. Using reminders appears to be effective in many medical fields [35,36,37]. Our study showed that recommended desexing age was associated with sending an appointment reminder the day before surgery was booked, with practices that recommended desexing from 21 to 25 weeks more likely to use this promotional step. There were also associations between actual age of desexing and some practice actions (discussing desexing with all new owners and sending reminders to have the pet desexed and/or the day before the booking) with practices that desexed, on average, at 21 or more weeks more likely to use these promotional steps.

Recommended ages for desexing were provided by 56% of practices on their website, on information sheets, or by way of advice from reception staff. This is consistent with the results from an earlier Australian study where only approximately half of the urban veterinary practices surveyed provided information on desexing of cats on their websites [28]. Why this is so low was not explored in the current study and little relevant previous research was found. It could be that veterinarians feel uncertain about the optimal age to desex cats, they possess inadequate computer and social media skills to adequately promote desexing, that they believe it is the responsibility of others to promote the optimal age for desexing (governments or welfare agencies), or that optimal age for desexng is already well understood in the community. However, there is evidence that on the contrary optimal age for desexng is not well understood in the community [32].

Less commonly used steps by practices were incentives, such as free microchipping (17%) or reduced pricing for the procedure if it occurred before puberty (21%). A few veterinarians in the survey wrote comments, such as “All desexing is done at a much cheaper rate than other surgery to encourage clients to desex their pets.” Another wrote “Desexing is already a discounted “community service.”

Respondents selected numerous reasons they believed people did not have their cats desexed prior to puberty. The most commonly perceived reason was cost. However, this is not in agreement with a study of Auckland residents, the majority of whom did not agree with the statement, “Cost is the biggest factor when making a decision to desex your animal” [29], or a study in the US that found that less than 6% of people with intact animals reported cost as a factor [7]. In contrast, another US study [13] found a positive association between household income and whether pet cats were desexed. This same positive association was also reported in a New Zealand-wide survey [38]. Therefore, the extent to which household income influences cat desex decisions remains unclear.

Simply forgetting about desexing or not getting around to it was selected by 68% of respondents. This seems a likely reason as the temporal window to desex a cat after its last vaccination and before puberty is quite short. The 2019 PAW annual report into animal welfare in the United Kingdom [39] found “haven’t thought about it” and “not got around to it yet” were among the most common three reasons given by owners of entire cats for not having their cat desexed (the most common reason was the cat “doesn’t go outside”). A range of other considerations were also identified including considerations of the cat’s welfare [39].

Other less commonly perceived reasons included “It is nice for their children to see the pet have a litter” (39%), “the litter can be sold” (26%), and the owner’s belief that they can confine the cat sufficiently to prevent a litter (27%). These considerations would be more difficult to address even with improved understanding of the benefits of prepubertal desexing. More general messaging about cat overpopulation could help. Cats are prolific breeders, as a female cat is able to produce at least two litters a year with an average of four kittens in each litter [40].

Although Australian veterinarians usually report that a high percentage of their cat clients are desexed [9], much lower percentages of cats admitted to RSPCA shelters across Australia have been desexed before admission. In one study, only 36% of all feline admissions were previously desexed (50% of adult cats and 22% of kittens,) and only 47% of owner-surrendered cats [11]. This large difference may be partly because many cats surrendered to shelters have never been to a veterinary clinic and so are not considered clients by veterinarians. This highlights the importance of considering cat populations as having quite distinct subsets, with cats potentially moving from one subset to another. For example, cats that are regularly taken to a veterinary clinic may be viewed as a separate sub-population of cats from those sub-populations from which most cats surrendered to shelters are drawn [11,12]. Further, there are clearly distinct subpopulations of cats that contribute surrenders, as while many are owner-surrendered cats, many others are surrendered as strays [11,12], and while 47% of owner-surrendered cats were desexed before surrender, only 24% of stray surrenders were desexed before surrender [11]. There is a need to clarify the relative contributions of these various sub-populations to the numbers of surrendered adult cats and kittens along with frequency of cat movements into and between these subpopulations, as this would define the likely impact of more veterinary practices recommending and conducting desexing before puberty on these numbers. In the interim, given the recommended and actual desexing ages along with the substantial proportion of practices where high percentages of cats desexed had already had a litter, it seems likely that important reductions would occur if more practices recommended and conducted most cat desexing before puberty.

## 5. Conclusions

More than 80% of respondents thought that private practice veterinarians have a very important role in preventing the birth of unwanted kittens. Almost all practices (98%) were taking active steps to promote desexing and most veterinarians considered those steps to be successful (67%) or very successful (27%). However, almost 45% of respondents recommend desexing of cats at the traditional age of 6 months, and more than 56% did not actually desex until cats were at least 6 months or more of age. Similarly, only 8% of respondents recommend desexing of cats at 16 weeks of age or less (a category where virtually all would be prepubertal), and even fewer, less than 5%, actually desexed on average at that age. This is consistent with earlier studies, and suggests that while it is apparent from those earlier studies that in Australia, veterinarians are generally more prepared to recommend prepubertal desexing than in New Zealand and the United Kingdom, the proportion of Queensland veterinarians who recommend and undertake prepubertal desexing is not high. The results from this study indicate that, in Queensland, recommended and actual desexing ages are commonly too late to ensure that cats are desexed prior to reaching reproductive age, and that female cats that are desexed have commonly already had a litter. There is a need to clarify the likely impact of more veterinary practices recommending and conducting desexing before puberty on numbers of unwanted cats and numbers surrendered to shelters. It seems likely that important reductions would occur if more practices recommended and conducted most cat desexing before puberty. Thus, it is important to increase the proportion of veterinarians both recommending and conducting desexing procedures on prepubescent cats.

A better understanding of the drivers of age at which cats are desexed, including why veterinarians are recommending particular ages, could inform the design of strategies to reduce numbers of unwanted cats. While one avenue of improving the proportion of cats that undergo prepubertal desexing is to increase the proportion of veterinarians both recommending and conducting these procedures, another avenue is to increase the number of owners who elect to have their cats desexed prior to puberty.

## Figures and Tables

**Table 1:** Ages that respondents’ recommended cats be desexed and actual average ages that cats are desexed in Queensland veterinary practices.

Age Category	Recommended Desexing Age for Cats (n = 255) ^1^	Actual Average Age That Cats are Desexed (n = 254) ^2^
8–10 weeks	3 (1%)	1 (0%)
11–12 weeks	4 (2%)	5 (2%)
13–16 weeks	12 (5%)	5 (2%)
17–20 weeks	41 (16%)	30 (12%)
21–25 weeks	61 (24%)	55 (22%)
6 months	114 (45%)	127 (50%) ^3^
9 months	0 (0%)	16 (6%)
Other	20 (8%)	15 (6%)

^1^ At what age do you recommend that cats are desexed? ^2^ What is the actual average age cats are desexed in your practice? ^3^ Includes 27 respondents that selected “over 25 weeks.”

**Table 2:** Ways in which Queensland veterinary practices promote desexing of cats.

Step	Number (Percentage of 244 Respondents)
Discuss desexing with new owners when they bring their kitten for vaccination	235 (96%)
Suggest desexing when new cat receives final vaccination	177 ^1^ (73%)
Suggest setting a desex date when the cat receives its final kitten vaccination	147 (60%)
Send a desexing reminder by email/sms/phone etc if an appointment is not made for new pet	169 (69%)
Send an appointment reminder the day before desexing surgery has been booked in	163 (67%)
Discuss desexing options with owners of all entire adult cats	204 (84%)
Have information about desexing on practice website	139 (57%)
Have recommended ages for desexing available: on website; on information sheets; and as advice from reception staff	136 (56%)
Regularly have information about desexing in practice newsletter	38 (16%)
Have posters promoting desexing in practice waiting room	51 (21%)
Offer incentives to clients (E.g. free microchipping at desexing)	41 (17%)
Offer reduced price for desexing cats before their first season	52 (21%)
Other ^1^	15 (6%)

^1^ The majority of these stated: participating in desexing promotions.

**Table 3:** Veterinarians’ opinions on reasons why clients do not have their cats desexed before puberty.

Reason	Number (Percentage of 243 Respondents)
They forgot or just didn’t get around to it	166 (68%)
They can’t afford it or think that they can’t	180 (74%)
They think it is too expensive	126 (52%)
They think their cat is too young	57 (23%)
They think the operation is too dangerous	30 (12%)
Vet has advised them to wait until their pet is older on medical grounds	3 (1%)
They think it best to allow the pet to have a litter before desexing	97 (40%)
They think they will make money selling the kittens	63 (26%)
They think it is nice for their children to see the pet have a litter	94 (39%)
They are opposed to desexing for religious or cultural reasons	14 (6%)
They think it is unnecessary as they can confine their cat	66 (27%)
Other	17 (7%)

**Table 4:** Actual average ages that cats are desexed in relation to recommended desexing age for 256 Queensland veterinary practices (bolded values are numbers of practices where recommended and actual categories are the same).

Recommended Desexing Age	Actual Average Age That Cats are Desexed					
	Upto 16 Weeks	17 to 20 Weeks	21 to 25 Weeks	Over 25 Weeks	Not Recorded	Total
Upto 16 weeks	**6**	10	2	1	0	19
17 to 20 weeks	0	**12**	15	12	2	41
21 to 25 weeks	0	1	**26**	28	6	61
Over 25 weeks	0	2	9	**100**	3	114
Not recorded	1	5	3	6	6	21
Total	7	30	55	147	17	256

**Table 5:** Percentages of 235 Queensland veterinary practices that used various steps to promote desexing of cats with respect to recommended desexing age for cats.

Step	Recommended Desexing Age				*p*
	Upto 16 Weeks (n = 19)	17 to 20 Weeks (n = 41)	21 to 25 Weeks (n = 61)	Over 25 Weeks (n = 114)	
Discuss desexing with new owners when they bring their kitten in for vaccination.	18 (95%)	38 (93%)	58 (95%)	104 (91%)	0.887
Suggest desexing when new cat receives final vaccination.	17 (89%)	38 (93%)	50 (82%)	88 (77%)	0.129
Suggest setting a desexing date when the cat receives its final kitten vaccination. ^1^					
Send a desexing reminder by email/sms/phone, etc if an appointment is not made for new pet.	11 (58%)	26 (63%)	46 (75%)	74 (65%)	0.356
Send an appointment reminder the day before desexing surgery has been booked in.	12 (63%)	28 (68%)	49 (80%)	65 (57%)	0.018
Discuss desexing options with owners of all entire adult cats.	18 (95%)	33 (80%)	47 (77%)	90 (79%)	0.399
Have information about desexing on the practice website.	12 (63%)	22 (54%)	35 (57%)	62 (54%)	0.899
Have recommended ages for desexing available: on website: on information sheets: and as advice from reception staff.	7 (37%)	19 (46%)	42 (69%)	61 (54%)	0.034
Regularly have information about desexing in practice newsletters.	2 (11%)	8 (20%)	14 (23%)	14 (12%)	0.253
Have posters promoting desexing in practice waiting room.	4 (21%)	7 (17%)	13 (21%)	24 (21%)	0.955
Offer incentives to clients (e.g., free microchip at desexing).	3 (16%)	12 (29%)	5 (8%)	19 (17%)	0.049
Offer reduced price for desexing cats before first season.	4 (21%)	8 (20%)	15 (25%)	23 (20%)	0.896
Other	2 (11%)	1 (2%)	1 (2%)	10 (9%)	0.125

^1^ Combined with, “Suggest desexing when new cat receives final vaccination,” for analyses.

**Table 6:** Percentages of 239 Queensland veterinary practices that used various steps to promote desexing of cats by actual average ages at which cats were desexed.

Step	Actual Average Age That Cats are Desexed				*p*
	Up to 16 Weeks (n = 7)	17 to 20 Weeks (n = 30)	21 to 25 Weeks (n = 55)	Over 25 Weeks (n = 147)	
Discuss desexing with new owners when they bring their kitten in for vaccination.	5 (71%)	27 (90%)	55 (100%)	134 (91%)	0.010
Suggest desexing when new cat receives final vaccination.	5 (71%)	28 (93%)	47 (85%)	120 (82%)	0.282
Suggest setting a desexing date when the cat receives its final kitten vaccination. ^1^					
Send a desexing reminder by email/sms/phone, etc if an appointment is not made for new pet.	1 (14%)	18 (60%)	42 (76%)	100 (68%)	0.010
Send an appointment reminder the day before desexing surgery has been booked in.	2 (29%)	15 (50%)	44 (80%)	94 (64%)	0.005
Discuss desexing options with owners of all entire adult cats.	5 (71%)	26 (87%)	47 (85%)	117 (80%)	0.546
Have information about desexing on the practice website.	4 (57%)	13 (43%)	30 (55%)	84 (57%)	0.581
Have recommended ages for desexing available: on website: on information sheets: and as advice from reception staff.	0 (0%)	12 (40%)	30 (55%)	88 (60%)	0.004
Regularly have information about desexing in practice newsletters.	0 (0%)	6 (20%)	5 (9%)	25 (17%)	0.330
Have posters promoting desexing in practice waiting room.	1 (14%)	3 (10%)	15 (27%)	29 (20%)	0.276
Offer incentives to clients (e.g., free microchip at desexing).	1 (14%)	5 (17%)	7 (13%)	24 (16%)	0.945
Offer reduced price for desexing cats before first season.	1 (14%)	9 (30%)	11 (20%)	27 (18%)	0.525
Other	1 (14%)	2 (7%)	1 (2%)	10 (7%)	0.244

^1^ Combined with, “Suggest desexing when new cat receives final vaccination,” for analyses.

## Appendix A

**Table A1:** AVA/RSPCA collaboration desexing survey.

Question	Response Options Offered
Q1 How important is the role of private veterinarians in preventing the birth of unwanted kittens?	Very important Somewhat important Little importance No importance
Q2 At what age do you recommend cats are desexed?	8–10 weeks 11–12 weeks 13–16 weeks 17–20 weeks 21–25 weeks 6 months 9 months Other (please specify)
Q3 What is the actual average age cats are desexed in your practice?	8–10 weeks 11–12 weeks 13–16 weeks 17–20 weeks 21–25 weeks Over 25 weeks 6 months 9 months Other (please specify)
Q4 Does your practice take active steps to promote desexing/remind clients to have their pet desexed?	Yes No Intermittently
Q5 If your practice promotes desexing of pet cats, which of the following, if any, do you do? Please tick as many as apply.	Discuss desexing with new owners when they bring their kitten in for vaccination. Suggest desexing when new kitten receives final vaccination. Suggest setting a desexing date when the cat receives its final vaccination. Send a desexing reminder by email/sms/phone, etc. if an appointment is not made for the new pet. Send an appointment reminder the day before surgery has been booked in. Discuss desexing options with owners of all entire adult cats. Have information about desexing on the practice website. Have recommended ages for desexing available: on website, on information sheets, as as advice from r3eception staff. Regularly have information about desexing in practice newsletters. Have posters promoting desexing in practice waiting room. Offer incentives to clients (e.g., free microchip at desexing). Offer reduced price for desexing cats before first season. Other
Q6 Which of the above options has worked best in your practice at ensuring desexing of cats occurs before sexual maturity?	Free response
Q7 How successful do you believe your approach is at getting clients to have their cats desexed?	Very successful Successful Not very successful Unsure
Q8 Why do you think people don’t have their cats desexed before puberty? (You may tick more than one.)	They forget or just don’t get around to it. They can’t afford it or think they can’t afford it. They think it is too expensive. They think it is too young. They think the operation is dangerous. You have advised them to wait until their pet is older on medical grounds. They think it best to allow the pet to have a litter before desexing. They think they will make money selling kittens. They think it is nice for their children to see the pet have a litter. They are opposed to desexing for religious or cultural reasons. They think it is unnecessary and they can stop litters through confinement. Other
Q9 Approximately what percentage of cats that you desex have had a litter?	0% 1–10% 11–25% 26–50% 51–75% 76–100%
Q10 If you have any other comments, please add them here.	Free response
Q11 Are you?	Male Female
Q12 In what age range are you?	<25 years 26–30 years 31–40 years 41–50 years 51–60 years >60 years
